# Autofluorescence Imaging of Parathyroid and Thyroid Under Visible and Near-IR Light Excitation

**DOI:** 10.3390/bios15060352

**Published:** 2025-06-03

**Authors:** Zhenguo Wu, Sam M. Wiseman, Haishan Zeng

**Affiliations:** 1 BC Cancer Research Institute, University of British Columbia, Vancouver, BC V5Z 1L3, Canada; zgwu@bccrc.ca; 2 Department of Surgery, St. Paul’s Hospital & University of British Columbia, Vancouver, BC V6Z 1Y6, Canada

**Keywords:** parathyroid detection, parathyroid autofluorescence, thyroid autofluorescence, near-IR autofluorescence imaging, visible autofluorescence imaging, thyroid surgery guidance, portable fluorescence imaging device

## Abstract

Identifying parathyroid glands during surgery is challenging and time-consuming due to their small size (3–5 mm) and camouflaged appearance in the background of the thyroid, lymph nodes, fat, and other neck structures. For the gland itself, it is also important to differentiate abnormal ones from normal ones. Accidental damage or removal of the normal glands can result in complications like hypocalcemia, which may necessitate lifelong medication dependence, and, in extreme cases, lead to death. The study of autofluorescence optical properties of normal and abnormal parathyroid glands and the surrounding tissue will be helpful for developing non-invasive detection devices. The near-infrared (NIR) autofluorescence characteristics of parathyroid and thyroid tissues have been studied extensively and are now used for parathyroid gland detection during surgery. Additionally, there have been a few reports on the UV-visible light-excited autofluorescence characteristics of these tissues with a focus on spectroscopy. However, there is a lack of high-resolution, side-by-side autofluorescence imaging comparisons of both tissue types under various excitation wavelengths, ranging from visible to NIR. We developed a standalone tabletop autofluorescence imaging system to acquire images of ex vivo specimens in the operating room under different excitation wavelengths: visible 405 nm, 454 nm, 520 nm, 628 nm, and NIR 780 nm. Autofluorescence imaging features of parathyroid adenomas for each excitation wavelength were described and compared. It was found that visible light excites much stronger autofluorescence from parathyroid adenoma tissue compared to NIR light. However, NIR excitation provides the best intensity difference/contrast between parathyroid adenoma and thyroid tissue, making it optimal for differentiating these two tissue types, and detecting parathyroid adenoma during surgery. The high fluorescent site under the NIR 780 nm excitation also generates high fluorescence under visible excitation wavelengths. Heterogeneous fluorescence patterns were observed in most of the parathyroid adenoma cases across all the excitation wavelengths.

## 1. Introduction

Pathologies relating to neoplasia and functional changes of the parathyroid (PT) and thyroid (TH) have led to operations often being performed on these glands, which are the most common neck surgeries in North America, with over 20 million individuals affected. Four PT glands are located on the posterior surface of the TH. During surgery, the normal PT glands need to be preserved, while the abnormal ones need to be found and removed. These glands can be difficult and time-consuming to identify because of their small size (3–5 mm) and their camouflaged appearance in the presence of adjacent thyroid, lymph nodes, fat, and other neck structures. The normal PT glands may be accidentally damaged or mistakenly removed during surgery, which can lead to issues such as hypocalcemia, which may necessitate lifelong medication dependence, and, in extreme cases, lead to death.

Therefore, there is a pressing need for a device that can help the surgeon to reliably identify the PT glands or even differentiate the normal and the abnormal PT glands. The study of natural optical properties of both normal and abnormal PT and TH tissues can be helpful for developing such non-invasive detection devices.

There is strong evidence in the literature that the PT glands have near-IR (NIR) autofluorescence properties that are distinct from TH and other surrounding normal tissues [1,2]. When excited by light with a center wavelength of approximately 780 nm, parathyroid glands generate an autofluorescence signal that is peaked at approximately 820 nm. The signal intensity of the parathyroid is reported to be 3–5 times higher than the surrounding tissues [2]. Both laboratory developed devices [2,3,4,5,6] and commercial imaging systems [7] have been designed to assist with the detection of parathyroid glands during surgery based on their NIR autofluorescence properties. In recent years, researchers have started to explore the NIR autofluorescence of abnormal PT glands, and it was found that parathyroid adenomas generate heterogenous NIR autofluorescence pattern mostly with a high fluorescence portion corresponding to the normal parathyroid tissue [8].

Only a few studies have reported on the UV and visible autofluorescence properties of PT and TH with a focus on spectroscopy. Pitman et al. studied the autofluorescence spectral properties of thyroid tissue under 300 nm and 320 nm excitation [9]. Giubileo et al. measured the excitation–emission matrix of healthy and cancerous thyroid tissue in a wavelength range of 200 nm to 700 nm [10]. However, their study specimens were stored in formaldehyde before being transported to the lab for fluorescence spectral measurements. This type of sample treatment would have significantly altered the tissue molecular structures and, consequently, the tissue fluorescence properties, rendering the results inapplicable for clinical applications. Brandao et al. studied the optical properties of parathyroid tissues, including the absorbance, the fluorescence spectral spectroscopy, and the fluorescence lifetime at eight different excitation wavelengths covering UV and visible range [11]. The parathyroid tissue displayed varied autofluorescence peak positions for the eight excitation wavelengths (285, 290, 300, 365, 410, 525, 575, and 600 nm). These studies provided only spectral data for either thyroid or parathyroid tissue. They do not include imaging data or comparative results for the two types of tissue.

Here, we developed a standalone tabletop autofluorescence imaging system that can acquire high resolution images of ex vivo specimens in the operating room under different excitation wavelengths, from visible (405 nm, 454 nm, 520 nm, 628 nm) to NIR 780 nm. Since parathyroid adenoma is the most common type of tumor that can develop in the parathyroid glands, in this study, we aimed to use the system to evaluate the high-resolution autofluorescence imaging properties of surgically removed parathyroid adenomas and thyroid lobes under the excitation of visible and NIR light. The images also provided a direct comparison of the autofluorescence intensity differences between the two types of tissue for each excitation wavelength.

## 2. Materials and Methods

### 2.1. System Setup

**Figure 1:**
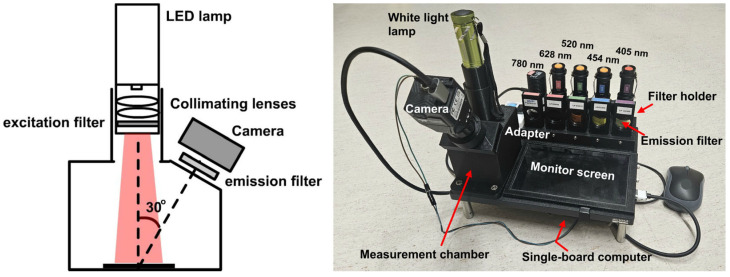
Schematic configuration (**left**) and photograph of the autofluorescence imaging system (**right**). A white-light LED lamp is used for color imaging, while 5 different narrow band LED lamps are used for autofluorescence imaging at different excitation wavelengths, covering broad visible and NIR wavelength range (405 nm, 454 nm, 520 nm, 628 nm, and 780 nm).

We built an autofluorescence imaging system to evaluate the surgically removed tissue specimens, as shown in Figure 1. The imaging system includes an excitation light source (the LED lamp), a specimen chamber, and a CMOS camera. In the light source, the LED lamp is collimated and filtered by an excitation filter to purify the excitation wavelength band. An emission filter was inserted before the camera to block the reflected excitation light and pass through the longer wavelength fluorescence light emitted by the tissue specimen. The angle between the excitation optical path and the imaging detection optical path was set at 30° degrees.

**Figure 2:**
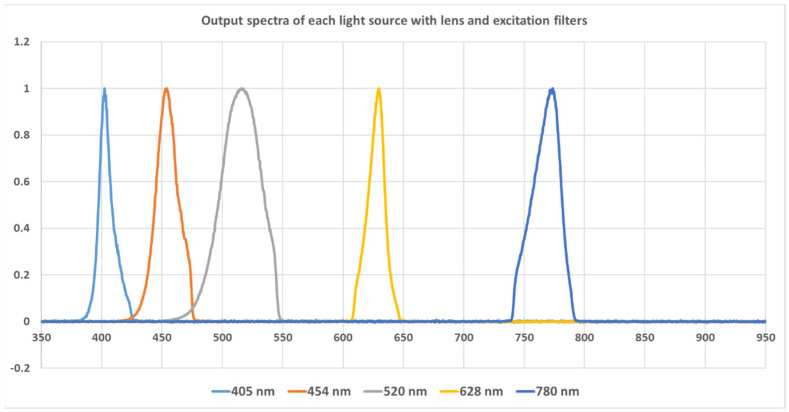
The emission spectra of the five lamps after the excitation filters.

The system allows us to acquire autofluorescence images of the tissue sample under five different excitation wavelengths (405 nm, 454 nm, 520 nm, 628 nm, and 780 nm). Five LED lamps were used alternatively for this purpose; they are referred to as LED 1–LED 5 in the following description. LED 1 to LED 4 were visible-light narrow-band LEDs obtained from Amazon, with emission wavelengths of 405 nm, 454 nm, 520 nm, and 628 nm, respectively. They are collimated by a built-in plastic lens and another achromatic doublet lens (f = 30 mm, model # AC254-30A, Thorlabs, Newton, NJ, USA). LED 5 is a narrow-band NIR LED, with a central wavelength of 780 nm (M780L3—780 nm, Thorlabs). It is collimated using an aspheric condenser lens (f = 20.1 mm, ACL2520U-B, Thorlabs). Each lamp was paired with an excitation filter to provide spectrally purified excitation light. The five corresponding excitation filters are as follows: 425 nm short-pass filter (#84-703, Edmund Optics, Barrington, NJ, USA), 475 nm short-pass filter (#84-705, Edmund Optics), 550 short-pass filter (#84-708, Edmund Optics), 628 nm band-pass filter (# 84-103, Edmund Optics, 32 nm bandwidth), and 769 nm band-pass filter (#84-105, Edmund Optics, 41 nm bandwidth). The spectra of the 5 LED light sources after the corresponding excitation filters are shown in Figure 2. All the lamps are powered by batteries. The output power of the five lamps was measured to be 150 mw, 120 mw, 110 mw, 115 mw, and 240 mw, respectively. The five corresponding emission filters are GG455 absorption glass long-pass filter (3 mm thickness, 455 nm cut-on wavelength, model #45-068, Edmund Optics), GG495 long-pass (3 mm, 495 nm cut-on wavelength, #32-753, Edmund), OG590 long-pass (3 mm, 590 nm cut-on wavelength, #46-063, Edmund Optics), RG695 long-pass (3 mm, #32-756, Edmund Optics), and combined RG830 long-pass (3 mm, 830 nm cut-on wavelength #32-758, Edmund Optics) and 832 nm band-pass filter (#84-091, Edmund Optics, 45 nm bandwidth). The last emission filter is an integration of an absorption glass long-pass filter and an interference filter. The interference filter was used to block most of the excitation light, while the absorption filter was used for absorbing the scattered large-angle stray light.

In addition, a white-light LED lamp was also included to take reflectance color images of the tissue specimens. In this case, no excitation filter or emission filter was used.

A CMOS color camera (LT-UC00-C2050, Teledyne Technologies, Thousand Oaks, CA, USA) was used to take both color images and fluorescence images, with its IR filter removed to allow the detection of NIR signals (700–1000 nm). The CMOS sensor is sensitive to light in the range of 400 nm to 1000 nm. R, G, and B channels have similar sensitivities in the wavelength range of 800 nm–1000 nm. For autofluorescence imaging, signals from all three channels are added together to generate a gray-scale image reflecting the integrated fluorescence intensity over a broad wavelength range. A 3D-printed adapter was designed to attach the camera to the measurement chamber. The camera adapter has a slot to hold the emission filter. Whenever the excitation lamp is changed, the corresponding emission filter, that is mounted in a filter holder, is picked up and inserted into the camera adapter so that the corresponding autofluorescence image can be taken. A small single-board computer (LattePanda V1, Zhiwei Robotics Corp., Shanghai, China) with a 7-inch IPS display screen was used to control and manage the imaging system. A custom desktop application was programmed to acquire and save images. A battery power bank was used as the power supply. The whole system was designed to be a standalone system for convenient bedside use in the operating room.

### 2.2. Ex Vivo Specimen Evaluation

The system was developed at the BC Cancer Research Institute, and the ex vivo measurements were made in the operating room at St. Paul’s Hospital, Vancouver, Canada in close collaboration with an endocrine surgeon (SMW). The study was approved by the UBC-Providence Health Care Research Ethics Board of the University of British Columbia (Certificate # H22-02129), and informed consent was obtained from all study participants. Patients undergoing thyroid or parathyroid operations in the surgical practice of SMW, a high-volume thyroid/parathyroid surgeon (>300 thyroid and/or parathyroid operations annually) who practices in a tertiary Endocrine Surgical referral center (St. Paul’s Hospital) in Vancouver, BC, Canada, were eligible for study enrolment.

For all cases, the operating surgeon wore 2.5× magnification loupes and utilized intraoperative recurrent laryngeal nerve monitoring. For parathyroid operations, a focused procedure that employed intraoperative PTH measurement was the standard approach for cases localized preoperatively. The surgically removed parathyroid adenoma or thyroid lobes were handed off from the surgical field and placed in a small black disposable dish, to be evaluated with the autofluorescence imaging system, before being placed into formalin and sent to pathology for routine histopathological assessment.

The surgical specimen, along with the dish, was then placed into the measurement chamber of the imaging system, which prevented contamination with ambient light, so that the operating room workflow was minimally affected. A white-light image and five autofluorescence images were sequentially acquired with the corresponding lamp and emission filter. For all the measurements, the gain of the camera was set to 10 and the exposure time was initially preset for each lamp to 7 ms (white light), 125 ms (LED 1), 288 ms (LED 2), 194 ms (LED 3), 231 ms (LED 4), and 1300 ms (LED 5). Depending on the actual intensity of the image, the exposure time was adjusted accordingly. Both the specimen’s anterior and posterior surfaces were imaged.

## 3. Results

A total of nine parathyroid adenomas and seven thyroid lobes (four goiters, one follicular adenoma, one papillary thyroid cancer, and one medullary thyroid microcarcinoma) from fifteen patients were evaluated. Seventeen parathyroid adenoma faces and thirteen thyroid lobe faces were imaged. Study patient preoperative, intraoperative, and postoperative characteristics are summarized in Table 1.

**Figure 3:**
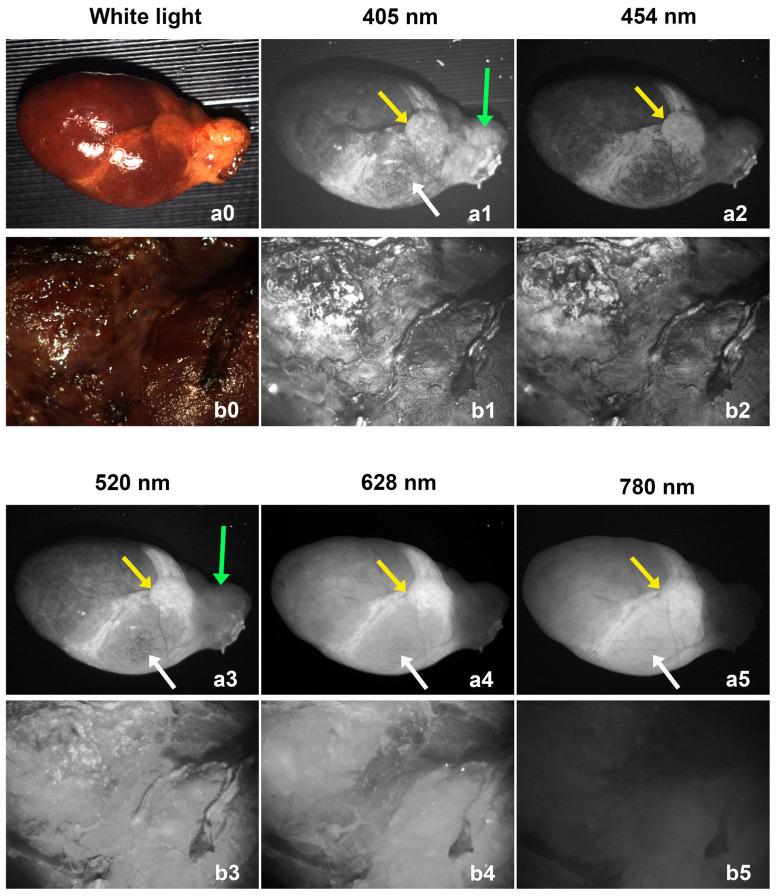
The autofluorescence images of a parathyroid adenoma (**a0**–**a5**) from case 3 and a thyroid lobe (**b0**–**b5**) from case 8 under the excitation of different wavelengths. The green arrows point to the site where the fluoresence is strong under 405 nm and 520 nm but weak under other wavelengths. The white arrows point to the site where the fluorescence is strong under 405 nm, 520 nm, 628 nm and 780 nm but weak under 454 nm. The yellow arrows point to the site where the fluorescence is strong under all excitation wavelengths.

Both parathyroid adenoma and thyroid lobe showed autofluorescence for all the selected excitation wavelengths (405 nm, 454 nm, 520 nm, 628 nm, and 780 nm light). Sample images from a parathyroid adenoma and a thyroid lobe are shown in Figure 3. The upper row (a0–a5) shows images of a parathyroid adenoma, and the lower row (b0–b5) shows images of a thyroid lobe. The thyroid lobe is generally much larger than the parathyroid adenoma, which is why only the central portion of the lobe was imaged. The intensity of all images was normalized to the camera gain and exposure time for comparison purposes. For each wavelength, the illumination power was the same for imaging the parathyroid adenoma and thyroid lobe, and with the same camera gain and exposure time, we were able to visually compare the autofluorescence intensities between the parathyroid and thyroid tissues.

Different parts of the parathyroid adenomas exhibited different autofluorescence properties that depended on their excitation wavelength. What appeared to be fat located over the gland hilum covering the blood vessels (green arrows) had relatively strong fluorescence under 405 nm and 520 nm light excitation, but low fluorescence under 454 nm, 628 nm, and 780 nm excitation. The bottom region (white arrows) had relatively strong fluorescence under 405 nm, 520 nm, 628 nm, and 780 nm excitation. The middle region (yellow arrows) has strong fluorescence for all excitation wavelengths. This area may represent the normal parathyroid gland remnant that the adenoma evolved from.

The thyroid lobe exhibited relatively strong autofluorescence for the four visible excitation wavelengths (405 nm, 454 nm, 520 nm, and 628 nm), but weak autofluorescence for the 780 nm NIR excitation. From these images, we can tell that the 780 nm NIR excitation gives the best image contrast (largest fluorescence intensity difference) between parathyroid and thyroid tissues.

**Figure 4:**
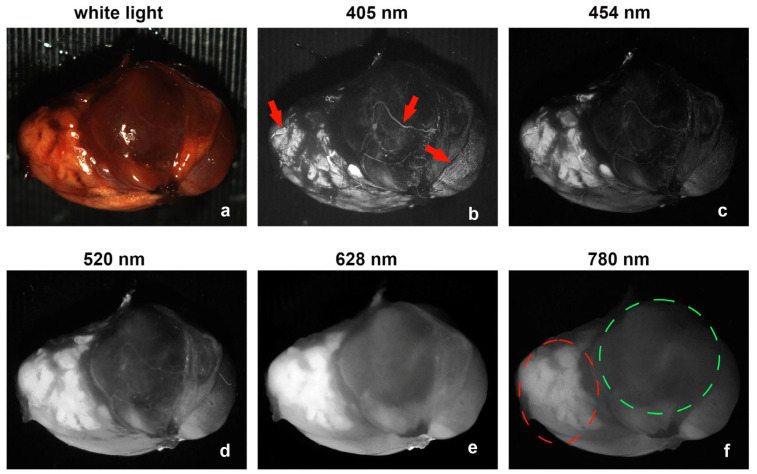
The autofluorescence images of a parathyroid adenoma from case 9 under different excitation wavelengths show heterogeneous patterns. The wavelengths of the excitation light are labeled on the top of each image: (**a**) white light, (**b**) 405 nm, (**c**) 454 nm, (**d**) 520 nm, (**e**) 628 nm and (**f**) 780 nm. The red arrows in (**b**) point to high fluorescent regions under 405 nm excitation. The red and green dotted circles in (**f**) outline the corresponding high and low fluorescent regions under 780 nm excitation.

A heterogeneous autofluorescence pattern was also observed in some cases, as shown in Figure 4. The pattern can be observed for all excitation wavelengths. The high-fluorescent and low-fluorescent regions are outlined by a dashed red circle and a green circle, respectively, in Figure 4f. The high-fluorescent region can also be seen in Figure 4a–e. The heterogeneous pattern exhibited by the parathyroid adenoma could be related to its hyperfunctioning characteristics [8,12]. Figure 4b shows some features with high contrast under 405 nm excitation, as indicated by red arrows, which are not clearly visible in Figure 4a,d–f. The corresponding biological structures deserve further investigation.

**Figure 5:**
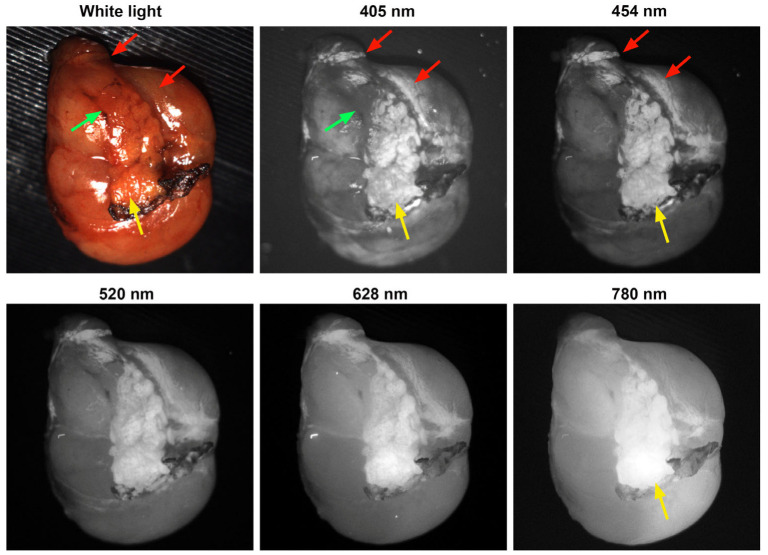
The autofluorescence images of a parathyroid adenoma from case 4 under different excitation wavelengths. The wavelength of the excitation light was labeled on top of each image.The red arrows point to areas that have high fluorescence under 405 and 454 nm excitation but not clearly distinguishable under white light imaging. The green arrows point to a site that has low fluorescence under 405 nm excitation and not distinguishable under white light imaging. The yellow arrows point to a region that has high fluorescence under all excitation wavelengths.

Another parathyroid imaging example is shown in Figure 5. The entire gland exhibits relatively high autofluorescence intensity under 780 nm excitation, while its middle part (yellow arrows) has extremely high intensity. As discussed in Figure 3, the middle area could potentially represent the normal parathyroid gland remnant. The high autofluorescence nature of this area exists for all the excitation wavelengths. This may imply that the fluorophores that can help to distinguish parathyroid from the surrounding tissue can be efficiently excited by light of a broad range of wavelengths, from 405 nm to 780 nm. Compared to white-light imaging, autofluorescence imaging provides better contrast for distinguishing different tissue components of the resected gland. The green arrows and red arrows point to areas of improved contrast of autofluorescence imaging under visible light excitation.

The autofluorescence intensity of the parathyroid adenoma and thyroid lobe can also be compared numerically for each excitation wavelength because the light power, gain, and exposure time can be normalized to the same value. We performed a statistical analysis. For each image, the average fluorescence intensity was calculated as the average pixel intensity from an ellipse region selected from the highest-fluorescent area of the image. The same ellipse region is applied to all images that are acquired from the same specimen face. This is possible because, for each specimen, images were acquired sequentially without moving it, making them roughly co-registered, as shown in Figure 3, Figure 4 and Figure 5. For images that are acquired with an exposure time that is different from the preset value, the average pixel intensity was adjusted by multiplying the ratio (ratio = preset exposure time/actual exposure time).

**Figure 6:**
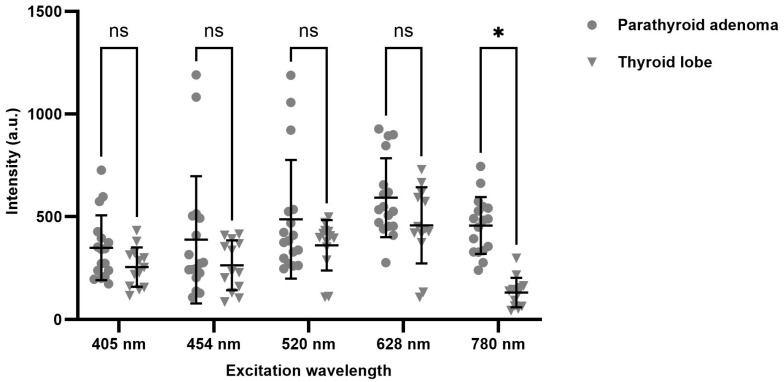
Comparison of the autofluorescence intensities of the parathyroid adenoma and thyroid lobe under different excitation wavelengths. ns indicates not significant difference (*p* > 0.05) and * indicates significant difference (*p* < 0.05) for the Mann-Whitney test.

Figure 6 shows a summary of the results. Each dot represents one measurement from one side of a parathyroid adenoma and each triangle represents one measurement from one side of a thyroid lobe. The mean value, the standard deviation, and the *t*-test result were plotted for each data group. For excitation wavelengths from 405 nm to 628 nm, there is a relatively large overlap between the autofluorescence intensity distribution of parathyroid adenoma and that of thyroid lobes. On the contrary, significant autofluorescence intensity differences were observed between the parathyroid adenoma and that of thyroid lobes when using 780 nm excitation.

## 4. Discussion

The study of the autofluorescence properties of parathyroid and thyroid tissues is helpful for the development of non-invasive detection devices to assist surgery. The NIR autofluorescence characteristics of parathyroid tissue have been studied for more than a decade and are now used to assist surgeons with parathyroid gland detection during surgery. A recent meta-analysis that included seven randomized clinical trials (1437 patients) reported that the use of camera-based NIR autofluorescence technology decreases the risk of postoperative hypocalcemia, inadvertent parathyroid resection, and permanent hypoparathyroidism [13]. However, there is currently a lack of high-resolution autofluorescence images of either normal or abnormal parathyroid glands despite a variety of laboratory-developed and commercial NIR imaging devices having been reported. UV-visible light excited autofluorescence properties of thyroid and parathyroid were studied by several groups, but high-resolution autofluorescence images of the two tissue types have not been reported either. We developed a stand-alone fluorescence imaging system to acquire high-resolution images of ex vivo samples under a broad range of excitation wavelengths of visible 405 nm, 454 nm, 520 nm, 628 nm, and NIR 780 nm in the operating room. Fresh resected specimens from the most common type of parathyroid tumor (parathyroid adenoma) were imaged and analyzed. The images are compared with those of thyroid lobes acquired from thyroid lobectomy. Both types of tissue generate autofluorescence signals for all the excitation wavelengths with various intensities. Autofluorescence imaging shows better contrast compared to white-light imaging to discern different tissue structures. Both 405 nm and 454 nm light can excite tissue fluorophores in the superficial layers, making fibrous connective tissue clearly visible. Light at 405 nm also generates autofluorescence in lipid tissue. Light at 520 nm and 628 nm penetrates deeper into the tissue, and the corresponding images show less detailed morphology structures. Light at 780 nm is within the optical window of biological tissue and penetrates the deepest into tissue. The images generated at this wavelength give the least details. Excitation at 780 nm provides the best contrast/statistically significant intensity difference between parathyroid adenomas and thyroid, while no statistically significant differences are observed under other excitation wavelengths. The heterogeneous fluorescence pattern was obviously observed in 12 of the 17 measurements under all excitation wavelengths. This heterogeneous pattern makes it more complex to calculate the average autofluorescence intensity. We selected the region with the highest fluorescence to carry out the calculation because it could be the region that will be used to distinguish parathyroid from the more homogenous thyroid tissue.

For all the measurements, a region with the highest autofluorescence under 780 nm excitation also shows the highest autofluorescence under excitation at other wavelengths. This implies that the fluorophore that distinguishes parathyroid tissue may have such an optical property that it can be efficiently excited by light in a broad range of wavelengths at least from 405 nm to 780 nm.

In this study, the fluorescence excitation efficiency was not compared among different wavelengths because the total intensity of the autofluorescence signal is difficult to estimate based on our setup. The fluorescence spectra were not measured, and the camera has different responses for different wavelengths. However, if we combine the camera’s sensitivity and the specimen’s excitation efficiency together as the excitation–detection efficiency (α), we can roughly estimate the value for each wavelength according to the following formula: average pixel intensity = α × power × exposure time × gain. The average α values (normalized to 520 nm) for each wavelength is calculated to be 0.81, 0.49, 1, 0.98, and 0.06. This suggests that the autofluorescence intensity of the most fluorescent part of the parathyroid adenoma could be much higher when excited under 405–628 nm than under 780 nm, considering that the IR response of the camera at 820 nm is at least about half of that for visible light. In the future, the excitation–emission matrix could be measured to give us a more comprehensive understanding of the fluorescence properties of parathyroid and thyroid tissues.

The imaging system currently is only suitable for ex vivo specimens; limited by this, only parathyroid adenomas are evaluated in this study because they can be easily collected from surgery compared to normal glands. Despite this, the high fluorescent part from adenomas may be attributed to normal remit which indirectly provides insight into the optical properties of normal glands. This was supported by Demarchi. et al.’s study, which shows that the high fluorescence “cap” of the parathyroid adenomas was actually normal glands tissue confirmed by histological examination [8]. The auotofluorescence imaging properties discussed in this paper are only from the gland surface. For internal tissue structures, the specimen needs to be further processed, e.g., cut in half, and these procedures are not included in the current ethical approval. Nevertheless, understanding the surface properties is still helpful for future development of non-invasive in vivo imaging devices. In addition, the observations of the current study are limited by its small patient population, that it was single surgeon/center based. Despite these limitations, in this study, NIR autofluorescence shows the most promise as an adjunctive technology to assist surgeons for intraoperative parathyroid gland identification.

From another aspect, the standalone tabletop fluorescence imaging system, equipped with multiple excitation wavelengths, is battery-powered, portable, reliable, cost-effective, and convenient to use. Its utility can be easily expanded beyond parathyroid and thyroid tissues, making it a potentially valuable tool for studying the autofluorescence imaging properties of various biological tissues in both clinical and laboratory settings.

## Figures and Tables

**Table 1:** Study patient preoperative, intraoperative, and postoperative characteristics.

CASE #	Age	Sex	Operation Performed	Surgical Indication	Preop US	Preop CTMIBI	Preop DECT or 4D CT	Preop Calcium (mmol/L)	Preop Ionized Calcium (mmol/L)	Preop PTH (pmol/L)	Final Pathological Diagnosis	Adenoma Diameter (cm)	Postop Calcium (mmol/L)
1	70	M	PTx, IPTH	PHP	LOC	NOLOC	NA	2.72	1.5	5.1	Parathyroid Adenoma	1.2	2.31
2	37	F	PTx	SC	NA	NA	NA	NA	NA	NA	Papillary Carcinoma	NA	NA
3	60	M	PTx, IPTH	PHP	LOC	LOC	NA	2.74	NA	13.3	Parathyroid Adenoma	1.5	2.38
4	57	F	PTx, IPTH	PHP	LOC	LOC	NA	3.01	NA	18.1	Parathyroid Adenoma	1.3	2.33
5	18	F	TL	GO	NA	NA	NA	NA	NA	NA	Goiter	NA	NA
6	40	F	PTx, IPTH	PHP	LOC	LOC	NA	2.8	1.49	13.3	Parathyroid Adenoma	1.7	2.46
7	65	F	PTx, IPTH	PHP	LOC	NA	LOC	2.74	1.47	12.6	Parathyroid Adenoma	1	2.43
8	41	F	TL	GO	NA	NA	NA	NA	NA	NA	Incidental microMTC & Hasimoto’s	NA	NA
9	49	F	PTx, IPTH	PHP	NOLOC	LOC	NOLOC	2.66	NA	12.6	Parathyroid Adenoma	0.9	2.24
10	49	F	TL	GO	NA	NA	NA	NA	NA	NA	Goiter	NA	NA
11	56	F	PTx, IPTH	PHP	LOC	LOC	LOC	2.84	1.59	24.6	Parathyroid Adenoma	3.5	1.86
12	54	F	TL	SC	NA	NA	NA	NA	NA	NA	Follicular Adenoma	NA	NA
13	79	F	PTx, IPTH	PHP	NOLOC	LOC	NOLOC	2.57	1.35	9.7	Parathyroid Adenoma	1.6	2.35
14	52	F	TL	GO	NA	NA	NA	NA	NA	NA	Goiter	NA	NA
15	65	F	PTx, IPTH	PHP	LOC	LOC	NA	2.78	NA	6.3	Parathyroid Adenoma	1.7	2.43
16	43	M	TL	CA-COMP	NA	NA	NA	NA	NA	NA	Goiter	NA	NA

**Abbreviations:** CTMIBI, sestamibi scan with non-contrast computed tomography; DECT, dual-energy computed tomography; F, female; LOC, localized; PTx, parathyroidectomy; IPTH, intraoperative parathyroid hormone measurement; TL, thyroid lobectomy; M, male; microMTC, subcentimeter medullary thyroid cancer focus; NA, not applicable; NOLOC, not localized; PHP, primary hyperparathyroidism; Preop, preoperative; PTH, parathyroid hormone level; US, ultrasound. Normal ranges: total calcium 2.1–2.6 mmol/L; ionized calcium 1.12–1.31 mmol/L; parathyroid hormone < 7.0 pmol/L.

## Data Availability

The original contributions presented in this study are included in the article. Further inquiries can be directed to the corresponding authors.

## Abbreviations

The following abbreviations are used in this manuscript:

NIR	Near-infrared
PT	Parathyroid
TH	Thyroid
CTMIBI	Sestamibi scan with non-contrast computed tomography.
DECT	Dual-energy computed tomography
LOC	Localized
PTx	Parathyroidectomy
IPTH	Intraoperative parathyroid hormone measurement
TL	Thyroid lobectomy
microMTC	Subcentimeter medullary thyroid cancer focus
NA	Not applicable
NOLOC	Not localized
PHP	Primary hyperparathyroidism
Preop	Preoperative
PTH	Parathyroid hormone level
